# What’s “up”? Impaired Spatial Preposition Processing in Posterior Cortical Atrophy

**DOI:** 10.3389/fnhum.2021.731104

**Published:** 2021-12-01

**Authors:** Zubaida Shebani, Peter J. Nestor, Friedemann Pulvermüller

**Affiliations:** ^1^Cognition and Brain Sciences Unit, University of Cambridge, Cambridge, United Kingdom; ^2^Psychology Department, Sultan Qaboos University, Muscat, Oman; ^3^QLD Brain Institute, The University of Queensland, Brisbane, QLD, Australia; ^4^Brain Language Laboratory, Department of Philosophy and Humanities, WE4, Freie Universität Berlin, Berlin, Germany; ^5^Berlin School of Mind and Brain, Humboldt Universität zu Berlin, Berlin, Germany; ^6^Einstein Center for Neurosciences, Charité University Medicine Berlin, Berlin, Germany

**Keywords:** posterior cortical atrophy, PCA, working memory, semantic processing, embodiment cognition, spatial prepositions, spatial language processing, category specific impairments

## Abstract

This study seeks to confirm whether lesions in posterior regions of the brain involved in visuo-spatial processing are of functional relevance to the processing of words with spatial meaning. We investigated whether patients with Posterior Cortical Atrophy (PCA), an atypical form of Alzheimer’s Disease which predominantly affects parieto-occipital brain regions, is associated with deficits in working memory for spatial prepositions. Case series of patients with PCA and matched healthy controls performed tests of immediate and delayed serial recall on words from three lexico-semantic word categories: number words (*twelve*), spatial prepositions (*behind*) and function words (e.g., *shall*). The three word categories were closely matched for a number of psycholinguistic and semantic variables including length, bi-/tri-gram frequency, word frequency, valence and arousal. Relative to controls, memory performance of PCA patients on short word lists was significantly impaired on spatial prepositions in the delayed serial recall task. These results suggest that lesions in posterior parieto-occipital regions specifically impair the processing of spatial prepositions. Our findings point to a pertinent role of posterior cortical regions in the semantic processing of words with spatial meaning and provide strong support for modality-specific semantic theories that recognize the necessary contributions of sensorimotor regions to conceptual semantic processing.

## Introduction

Understanding how words and their meanings are represented and processed in the human brain has been a central topic in cognitive neuroscience. Evidence derived from a range of methodological approaches suggests that different, often wide-spread, brain regions contribute to the semantic processing of different types of words, depending, at least to some degree, on the meaning carried by the words. For example, functional neuroimaging studies have found that words related to sounds activate auditory brain areas in left superior and middle temporal gyrus (Kiefer et al., 2008), words referring to actions activate motor regions in the fronto-central cortex (Hauk et al., 2004; Kemmerer and Gonzalez-Castillo, 2010; Shtyrov et al., 2014; Grisoni et al., 2016), words pertaining to colours engage temporal regions of the brain (Martin et al., 1995; Pulvermüller and Hauk, 2006; Simmons et al., 2007) and words denoting spatial relations (i.e., spatial prepositions) activate left inferior parietal regions (Damasio et al., 2001; Noordzij et al., 2008). These differential activations in sensory and motor brain regions suggest the involvement of action and perception systems in the processing of different semantic word categories. Findings from neuroimaging are supported by behavioural investigations showing motor-language interaction effects when subjects are required to process action-related language while engaged in motor activity (Boulenger et al., 2006; de Vega et al., 2013; Shebani and Pulvermüller, 2013, 2018). For example, Shebani and Pulvermüller (2018) have shown that performing movements with the hands and feet has a causal influence on the processing of action words by differentially impairing or enhancing working memory for arm- and leg-related action words depending on movement type. These studies demonstrate the functional relevance of sensorimotor systems to semantic processing.

In the views of some researchers, more convincing evidence for the important contributions of sensorimotor regions to semantic processing is the selective word processing deficits found in patients with focal brain lesions. Over the years, numerous neuropsychological investigations have targeted category-specific semantic processing. Much of this earlier work focused on dissociations between the categories of living and non-living things (e.g., Warrington and Shallice, 1984; Warrington and McCarthy, 1987) and nouns and verbs (e.g., Damasio and Tranel, 1993; Bak and Hodges, 1997, 2004; Neininger and Pulvermüller, 2003; Cotelli et al., 2006). More recently, studies have emphasized the important role of motor systems in action word processing as evidenced from patients with progressive brain diseases. For example, patients with Parkinson’s disease, a neurodegenerative disorder largely affecting the motor system, have been found to be impaired in processing action related language (Cotelli et al., 2007; Boulenger et al., 2008; Fernandino et al., 2013). Similarly, deficits in processing action words and concepts have been found in patients with motor neuron disease, a degenerative condition characterised by atrophy in motor and premotor cortex (Bak et al., 2001; Hillis et al., 2006; Grossman et al., 2008). Selective action word deficits have also been documented in cases of Semantic Dementia (SD), the temporal variant of frontotemporal dementia. SD, a degenerative brain disease originating in the temporal poles and spreading from there to other areas of temporal cortex as well as inferior frontal cortex, was found to degrade face related action words such as “speak” and “chew” (Pulvermüller et al., 2010).

As the functional relevance of motor systems in the processing of words with action related meaning appears to be well-established by a range of neurological disorders, this prompts the investigation of category specific semantic processing in patients with lesions located in sensory regions of the brain. Thus far, only a handful of neuropsychological studies have examined the role of perceptual systems in processing specific semantic word categories (Pulvermüller et al., 2010; Bonner and Grossman, 2012; Trumpp et al., 2013; Shebani et al., 2017). For example, in order to assess whether auditory association cortex is of special importance for the recognition of words and objects related to sound, Trumpp et al. (2013) tested a patient with a focal lesion in left posterior superior and middle temporal gyrus on a variety of tasks including lexical decision and category fluency. They found that the patient was consistently impaired in the semantic processing of sound-related objects and words such as “bell”. Similarly, to assess whether the temporal lobe plays a necessary role in processing words with strong visual meaning, using a lexical decision task, Pulvermüller et al. (2010) tested case series of patients with SD, who have anterior temporal lobe atrophy, and found that SD patients were significantly more impaired on processing words referring to colour than on processing words referring to object form.

To further examine the role of different cortices in semantic processing, we carried out a previous study looking at semantic word category processing in patients with SD and patients with Posterior Cortical Atrophy (PCA), an atypical form of Alzheimer’s disease (Shebani et al., 2017). The patients were selected for the study specifically because of their relatively focal lesions—in the anterior temporal lobes in the case of the SD patients and in parieto-occipital regions in the PCA patients (Nestor et al., 2003, 2006). Using a lexical decision test, the two patient groups were assessed on words from a range of lexico-semantic categories. Our results showed a greater impairment in processing words referring to colour in the SD group, consistent with previous findings (Pulvermüller et al., 2010). Importantly, the study was the first to document an impairment of spatial preposition processing in patients with PCA, suggesting a key role of posterior parietal cortex in processing words with spatial meaning (Shebani et al., 2017). These results are in line with predictions of the “semantic topography” model, according to which, words draw on category specific semantic circuits distributed across different cortical regions (Pulvermüller, 1999, 2013).

As the category-specific word processing deficits reported in most previous studies, including Shebani et al. (2017) and Pulvermüller et al. (2010), were found when tests of lexical decision were employed, we set out to substantiate these findings by investigating whether selective impairments can be found when a different task and experimental paradigm is used. A number of studies have demonstrated the importance of semantic processing in working memory tasks (e.g., Loaiza et al., 2011; Shivde and Anderson, 2011; Rose et al., 2014; Loaiza and Camos, 2018). To date, however, semantic word category processing has not been probed in patients using a working memory paradigm. According to models of working memory, keeping verbal information in working memory requires the continuous refreshing of semantic representations through the mechanism of attentional refreshing (Cowan, 1999, 2005; Barrouillet and Camos, 2007; Camos et al., 2009), which operates in conjunction with but independently from articulatory rehearsal to refresh memory traces in verbal working memory tasks (Camos et al., 2011; Shivde and Anderson, 2011; Loaiza and Camos, 2018). If category-specific regions do indeed make a necessary contribution to semantic word processing, keeping a series of words from the same category in working memory would place high processing demands on category-specific regions. By using a working memory setup we would expect to find pronounced word category deficits in patients with lesions in these regions. Additionally, since working memory representations decay with the passing of time (Baddeley, 1986; Cowan, 1999, 2005; Fuster, 2015), we would expect memory deficits to be more pronounced if words must be kept over a delay period.

In the present study, we used a working memory paradigm to assess word category processing in PCA patients (a description of the syndrome is provided below). Case series of patients performed tests of immediate and delayed serial recall on words from three lexico-semantic categories: number words, spatial prepositions and function words. The aim was to examine whether a working memory task would also reveal a selective word processing deficit in PCA patients as was found in the lexical decision study reported in Shebani et al. (2017). According to the semantic topography model (Pulvermüller, 1999, 2013), word-form representations located in the perisylvian language cortex which are involved in processing all word types are also bound to category-specific semantic circuits distributed across additional brain areas. These semantic networks are specific to a semantic category and store information about the objects, properties and/or actions the words typically denote. Words with spatial meaning, therefore, would receive processing from parieto-occipital regions involved in the processing of visuo-spatial information in addition to recruiting perisylvian language cortex which is involved in the processing of all word stimuli. Based on this model, because PCA patients have deficits in space orientating/positioning and in processing spatial concepts arising from their lesions in parieto-occipital regions, they are expected to be impaired on processing spatial prepositions which describe the location or relationship of objects in space. If a selective word category deficit were found using a working memory task, it would strengthen the argument that the cortical area affected in PCA is pertinent for the processing of spatial prepositions.

Posterior Cortical Atrophy, an atypical form of Alzheimer’s Disease, is a relatively rare neurodegenerative condition characterized by a dramatic and relatively selective decline in visuospatial and visuoperceptual skills. The progressive neurodegeneration affects posterior regions of the brain, primarily parieto-occipital regions (Nestor et al., 2003; Crutch et al., 2012). Commonly reported neuropsychological deficits include alexia, agraphia, visual agnosia, left-right disorientation, simultanagnosia and optic ataxia (Benson et al., 1988; Ross et al., 1996; Tang-Wai et al., 2004; Charles and Hillis, 2005; McMonagle et al., 2006; Crutch et al., 2012). People with PCA typically experience initial symptoms in their 50s or early 60s, at a younger age than those with the more common amnestic form of Alzheimer’s Disease (Crutch et al., 2012). Due to their visuospatial deficits, PCA patients often misreach for objects and, when reading, have difficulties accurately tracking lines of text. Other cognitive functions such as language skills, executive functions and memory, especially episodic memory, often remain well-preserved in PCA (Benson et al., 1988; Freedman et al., 1991; Mendez et al., 2002; Kas et al., 2011). However, deterioration of memory and language skills in the late stage of the disease has been reported in some patients (Levine et al., 1993; McMonagle et al., 2006) and impaired memory functioning early in the course of the disease has also been reported (Crutch et al., 2017; Ahmed et al., 2018), including deficits in visuospatial working memory (Funayama et al., 2021).

Neuropathological findings show that PCA is most commonly caused by Alzheimer’s Disease (Renner et al., 2004; Tang-Wai et al., 2004; Alladi et al., 2007). However, other underlying causes of PCA include corticobasal degeneration, prion disease and dementia with Lewy bodies (Victoroff et al., 1994; Tang-Wai et al., 2003a, b; Renner et al., 2004; Crutch et al., 2017). The distribution of pathological changes in PCA can be distinguished from typical Alzheimer’s Disease by a higher density of neurofibrillary tangles in occipital and posterior parietal regions and a lower density in anterior regions such as the prefrontal cortex (Levine et al., 1993; Hof et al., 1997; Galton et al., 2000). Atrophy and hypometabolism in PCA are bilateral though often more pronounced in the right hemisphere (Freedman et al., 1991; Nestor et al., 2003). Atrophy in PCA appears to remain mostly focused on posterior brain regions even late in the disease (Kas et al., 2011; Firth et al., 2019).

Not surprisingly, the profound visuospatial deficits of PCA patients have received greater interest than other cognitive symptoms associated with the syndrome. Language and memory impairments in PCA, and especially deficits in word processing and verbal working memory, have received less attention. One study by Trotta et al. (2019) examined working memory processes in PCA and found an impairment of verbal working memory in PCA patients. To our knowledge, however, only one other previous study, apart from Shebani et al. (2017), focussed on the processing of words that rely on spatial cognition in individuals with PCA. Gonzalez et al. (2019) investigated the comprehension of words referring to units of measurement in eight patients with a clinical diagnosis of PCA. As words related to measurement units (e.g., gram, metre) are closely linked to quantity and number, it was expected that their processing would highly depend on representations of space and magnitude in the parietal lobe. Interestingly, their study revealed degraded knowledge of measurement units in the PCA patients, suggesting that the numerical and spatial deficits resulting from parietal atrophy in PCA affects the processing of unit terms and concepts. Findings from a lesion overlap study of patients with impaired spatial preposition knowledge provide further support for the involvement of parietal regions in processing spatial language (Tranel and Kemmerer, 2004). These patient studies are consistent with imaging results which show the activation of parietal areas during the naming of spatial prepositions (Damasio et al., 2001) and in response to spatial prepositions (Noordzij et al., 2008). Words referring to numbers, on the other hand, have been found to activate both parietal regions (Dehaene, 1996; Pinel et al., 2001) and premotor/precentral regions (Tschentscher et al., 2012). Therefore, as number word processing appears to also recruit more anterior regions not affected in PCA, patients with PCA are likely to show reduced memory performance on spatial prepositions compared to number words.

The present study was guided by three hypotheses. The first two hypotheses are based on: (a) primary areas of degeneration in PCA; (b) neuroimaging studies showing the activation of these brain regions during the processing of words denoting spatial relations; and (c) previous findings of impaired spatial preposition processing in patients with lesions in parietal and parieto-occipital regions. Hypothesis 1 was that PCA patients would be impaired relative to controls on remembering spatial prepositions. Hypothesis 2 was that PCA memory performance would be worse on spatial prepositions than on number words since number words may also rely on motor regions for processing. Hypothesis 3, based on theoretical models in which working memory representations decay over time (Baddeley et al., 1975; Baddeley, 1986; Cowan, 1999, 2005; Barrouillet et al., 2011), was that PCA memory performance on spatial prepositions would be more impaired in delayed serial recall as lesions in parieto-occipital regions may prevent the semantic representations of spatial prepositions from being refreshed, thereby not allowing the decay of memory traces to be counteracted during the delay period. Function words were included in the study as a control word category.

## Materials and Methods

### Participants

Ten patients (five male) with a clinical diagnosis of PCA took part in the experiment (mean age = 59.2, s.d. = 5.8; mean years of education = 14.4, s.d. = 3.4). All patients had a clinical profile and evolution consistent with Alzheimer’s disease being the cause of their PCA, although this was not confirmed with biomarkers. They were, however, each followed for at least 2 years and none developed features of dementia with Lewy bodies or corticobasal degeneration, and, none had a clinical evolution to suggest prion disease; the consensus paper on classification of PCA (Crutch et al., 2017) was not yet extant when this study was conducted but applying its algorithm retrospectively, all patients would fit the description of “PCA-pure”. The patients were the same as those who took part in our previous study (Shebani et al., 2017). All patients were native English speakers. Eight of the 10 patients were right-handed with an average laterality quotient (LQ) of 93.4% (s.d. = 14.2) and two were left-handed (mean LQ = −90%, s.d. = 14.1) from a reduced version of the Oldfield handedness inventory (Oldfield, 1971). All were recruited from the Department of Neurology at Addenbrooke’s Hospital, Cambridge. Table 1 provides demographic information and neuropsychological scores on the Addenbrooke’s Cognitive Examination—Revised (ACE-R) and Mini-Mental State Exam (MMSE).

**Table 1:** Individual demographic information and neuropsychological test performance for the PCA patients.

Patient	Age	Years of Education	MMSE (30)	ACE-R Total (100)	Attention and (18)	Memory (26)	Fluency (14)	Language (26)	Visuo-spatial (16)
GD	67	18	9	22	4	4	4	8	2
JB	56	11	23	72	13	18	10	24	7
JP	54	14	11	31	5	3	3	19	1
LE	61	13	15	48	10	11	2	19	6
MMa	62	21	25	71	17	17	6	24	7
RL	52	15	22	70	14	13	8	25	10
SM	65	13	23	74	15	18	11	26	4
CS	62	17	NT	NT	NT	NT	NT	NT	NT
JH	50	11	NT	NT	NT	NT	NT	NT	NT
MMc	63	11	NT	NT	NT	NT	NT	NT	NT
Mean	59.2	14.4	18.3	55.4	11.1	12.0	6.3	20.7	5.3
s.d.	5.8	3.4	6.5	21.8	5.0	6.4	3.5	6.3	3.1

*MMSE and ACE-R are reported as whole scores*.

*ACE-R = Addenbrooke’s Cognitive Examination—Revised; MMSE = Mini-Mental State Exam; NT = not tested*. *Attention and Orientation, Memory, Fluency, Language and Visuo-spatial are sub-sections of the ACE-R*.

Additionally, 12 neurologically healthy participants (five male) served as control subjects in the experiment. Controls were right handed, native English speakers and matched to the patients in laterality quotient (mean LQ = 91.8%, s.d. = 12.6%) and years of education (mean = 14.5, s.d. = 2.4). They were slightly older than the PCA patients (mean age = 66.4, s.d. = 3.0). All participants provided informed, written consent prior to their participation. Ethics approval was obtained from the Cambridge Local Research Ethics Committee.

**Figure 1:**
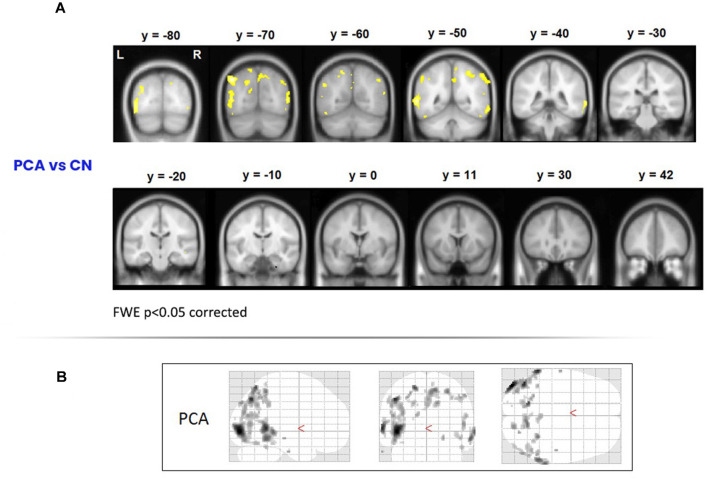
Areas of degeneration in the PCA group. **(A)** In a voxel-based morphometry study, PCA patients contrasted with controls (top panel) showed significantly reduced grey matter density in posterior brain regions. Family Wise Error correction was used (*p* < 0.05); therefore only most extreme areas of degeneration are shown. **(B)** Glass brains of the PCA patients showing degeneration in posterior cortical areas. PCA, Posterior Cortical Atrophy.

To assess areas of significant grey-matter degeneration in the PCA patients, a voxel-based morphometry (VBM) analysis was performed on MRI images acquired on a Siemens Trio 3T system. Healthy comparison data for this analysis were from 19 control participants. As shown in Figure 1, significant symmetrical degeneration in PCA was primarily observed in posterior parieto-occipital regions of both hemispheres.

Some of the more impaired patients were not able to complete the delayed serial recall block of the experiment. Therefore, the data of only 7 of the 10 PCA patients (three male; mean age = 58.3, s.d. = 5.7; mean years of education = 14.4, s.d. = 3.6; one left-handed patient, mean LQ of six right-handed patients = 93%, s.d. = 16.3) are included in the results of the delayed serial recall block of the experiment.

### Materials

Stimuli consisted of 72 lexical items divided into three different lexico-semantic categories of 24 words each: (1) Number words (e.g., *thirteen*, *hundred*), (2) Prepositions (e.g., *between, above*) and (3) Function words (e.g., *shall*, *yet*). Words from the Prepositions category referred to spatial relations. Function words were comprised mostly of conjunctions, interjections, pronouns and non-spatial prepositions. Table 2 provides a summary of the psycholinguistic and semantic variables for the stimuli including letter length, word frequency, bigram frequency, trigram frequency, number of neighbors, arousal, valence, imageability, concreteness and action relatedness.

**Table 2:** Psycholinguistic and semantic features of the word stimuli.

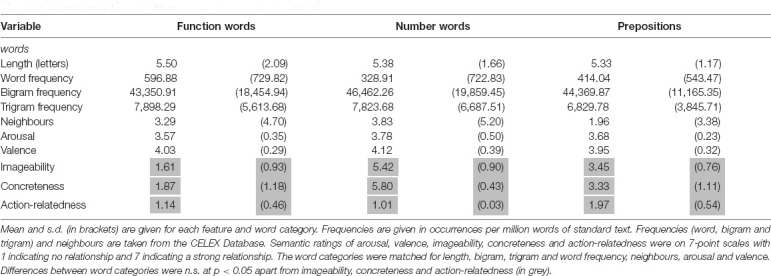	

### Procedure

In each trial, participants were presented auditorily with a string of 3–8 words from the same word category. Words were presented with an inter-stimulus interval of about 1 s, spoken by the examiner. Participants were required to keep the words in memory then repeat them in the same serial order in which they were presented when prompted to do so by the experimenter.

The number of words presented in each trial varied across participants and was determined separately for each participant before the experiment was started and based on each participant’s individual memory performance. The same list length was used for all categories for each participant. The list length was titrated to performance level so that word recall accuracy was between 60 and 70% for each participant. To keep accuracy at this level, PCA patients were presented with lists of three, four or six words. Six PCA patients performed the task with three word strings, two patients with four word strings and two patients with sox word strings (mean = 3.8, s.d. = 1.2). Adjusting list length for each individual participant was important to ensure that some errors were made during recall (~30% of trials). Healthy participants were able to manage longer lists and performed the memory task with either six word strings (nine controls) or eight word strings (three controls; mean = 6.36, s.d. = 0.8).

The experiment was run in two blocks consisting of 30–80 trials in total, depending on the number of words presented in each trial, with the full set of 72 words presented once in each block. Trials were randomized with the constraint that not more than two trials of words from the same category were presented consecutively. In the first block, immediate serial recall (ISR), participants were required to repeat the word string as soon as the last word was presented. In the second block, delayed serial recall (DSR), participants repeated the word string after a 5 s delay. The two blocks were run consecutively, counterbalanced across subjects in each group.

Instructions were given in writing and then repeated verbally. Questions were answered and there was sufficient opportunity for practice before starting the experiment, using stimuli different from those which were used in the experiment. The practice blocks served not only to familiarize participants with the task, but also to determine each participant’s memory performance level so that list lengths could be adjusted accordingly in the experimental blocks. Only after participants were comfortable with the task and after the experimenter identified their memory performance level was the experiment started. After every 10 trials, participants were asked if they would like a rest, and if so, a break was given. All performance was recorded and scored off-line.

### Statistical Analysis

Numbers of errors were calculated for each subject and for each of the three word categories (Function words, Number words and Prepositions) in ISR and DSR and submitted to statistical analysis. Since there were specific hypotheses, two-way ANOVAs were performed targeting the specific predictions motivating the study. A three-way ANOVA was not performed because of power issues due to the small set of subjects. To test differences in memory for the three word categories between the participant groups, a two-way repeated measures ANOVA (Group × Word Category) was performed. To test differences between performance in ISR and DSR on the three word categories, an additional ANOVA (Memory Task × Word Category) was performed. Planned Comparison F-tests were also conducted to reveal any statistical differences between word categories across and within participant groups. All tests used were two-tailed.

## Results

Average number of errors were calculated for each subject group and for each memory task. In ISR, PCA patients made an average of 24.7 errors (*SD* = 13.4, 34.3% errors) while control subjects made an average of 17.5 errors (*SD* = 8.2, 24.3% errors). When overall memory performance of the PCA patients on the memory task was compared with that of the control group, no significant differences were found (*F*_(1, 20)_ = 2.4, *p* = 0.14). This indicates that the ISR task was equally challenging for the two subject groups, which was expected as the paradigm was designed so that all participants made errors in ca. 30%. In DSR, PCA cases made an average of 31 errors (*SD* = 10.1, 43% errors) while controls made an average of 19.8 errors (*SD* = 7.9, 27.5% errors). F-tests revealed a significant difference between the PCA and control group in overall performance on the DSR task (*F*_(1, 17)_ = 7.2, *p* = 0.016), indicating that the DSR task was more challenging for the PCA patients.

The Analysis of Variance on memory performance on the three word categories across the two participant groups revealed a highly significant main effect of word category in both ISR (*F*_(2, 40)_ = 33.9, *p* < 0.0001) and DSR (*F*_(2, 34)_ = 48.4, *p* < 0.0001). The interaction of the Group and Word Category factors in the between group analysis failed to reach significance in ISR (*F*_(2, 40)_ = 2.2, *p* = 0.12), but was significant in DSR (*F*_(2, 34)_ = 3.9, *p* = 0.03). This interaction indicated that, in DSR, memory performance on the three word categories differed significantly between the PCA patients and healthy controls.

**Figure 2:**
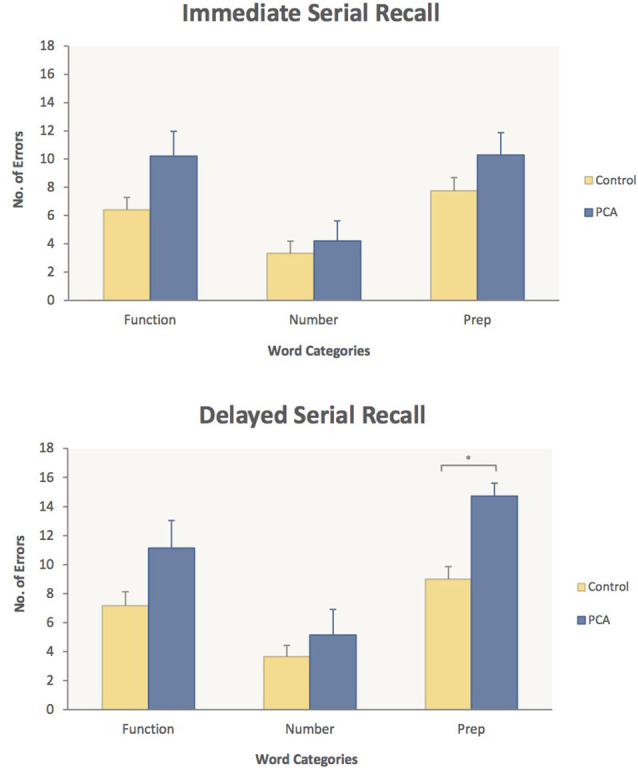
Performance on the working memory task in immediate serial recall (top panel) and delayed serial recall (bottom). Average number of errors and standard error measures are given for PCA patients (blue) and Control subjects (yellow) for each of the three word categories: Function words, Number words and Prepositions. PCA patients made significantly more errors on Prepositions compared to controls in the delayed serial recall task. This significant difference in performance is highlighted. *p < 0.05.

In ISR, the PCA patients’ poorest performance was on Prepositions [10.3 errors (PCA) vs. 7.8 (Control)] and Function words [10.2 errors (PCA) vs. 6.4 (Control)]. However, planned comparisons of the PCA patients vs. controls did not show any significant word category differences in ISR. In DSR, on the other hand, poor PCA performance on Prepositions relative to controls led to a highly significant word category difference [14.7 (PCA) vs. 9.0 (Control), *p* < 0.0005], indicating that, in the DSR task, PCA patients were significantly impaired relative to controls for Prepositions. Average errors made in each word category for the PCA patients and control subjects in each memory task are presented in Figure 2.

ANOVAs performed separately for each participant group yielded significant effects of word category in both memory tasks (all F values >15, all p values <0.0001). To test Hypothesis 2, planned comparisons were performed between word categories. As predicted, PCA patients made significantly more errors on Prepositions than on Number words in both ISR [10.3 vs. 4.2 errors (*F*_(1, 9)_ = 19.6, *p* < 0.002)] and DSR [14.7 vs. 5.1 errors (*F*_(1, 6)_ = 31.6, *p* = 0.001)]. PCA patients also made more errors on Prepositions than on Function words in DSR, but this difference did not reach significance [14.7 vs. 11.1 errors (*F*_(1, 6)_ = 3.7, *p* = 0.10)]. In the control group, significant differences were also found between Prepositions and Number words in ISR [7.8 vs. 3.3 errors (*F*_(1, 11)_ = 52.6, *p* < 0.001)] and DSR [9.0 vs. 3.7 errors (*F*_(1, 11)_ = 44.4, *p* < 0.001)]. All other word category differences were not significant.

**Figure 3:**
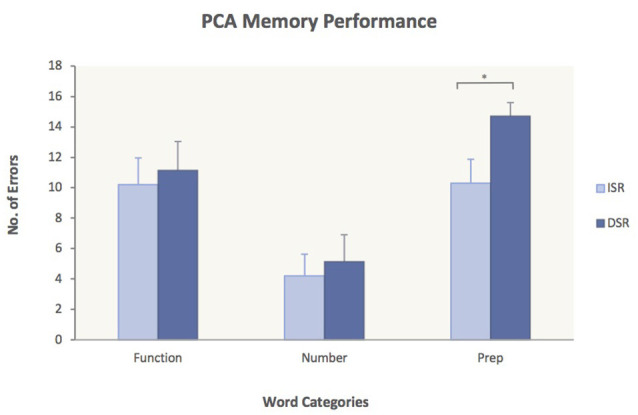
Performance of PCA patients on ISR compared to DSR. Average number of errors and standard error measures are given for each of the three word categories. PCA patients made significantly more errors on Prepositions in DSR compared to ISR. ISR, immediate serial recall; DSR, delayed serial recall. *p* < 0.05.

To test Hypothesis 3, ANOVAs were performed for each group with the factors Word Category and Memory Task (ISR/DSR). The analyses yielded significant main effects of word category for both subject groups [PCA: (*F*_(2, 30)_ = 32.4, *p* < 0.0001); Controls: (*F*_(2, 44)_ = 50.3, *p* < 0.0001)] but no significant interactions. Planned comparisons of the immediate vs. delayed serial recall task did not show significant differences in any of the word categories for the control group [Function words: 6.4 (ISR) vs. 7.2 (DSR), *p* = 0.21; Number words: 3.3 (ISR) vs. 3.7 (DSR), *p* = 0.52; Prepositions: 7.8 (ISR) vs. 9.0 (DSR), *p* = 0.68], indicating that the delay did not have an effect on memory performance for the healthy controls. In the PCA group, however, planned comparisons revealed significantly more errors on Prepositions in DSR compared to ISR [10.3 (ISR) vs. 14.7 (DSR), *p* = 0.046] while no significant differences were found for Function words [10.2 (ISR) vs. 11.1 (DSR), *p* = 0.73] or Number words [4.2 (ISR) vs. 5.1 (DSR), *p* = 0.68]. The significantly worse performance of PCA cases on Prepositions in DSR is presented in Figure 3.

## Discussion

The present study used a working memory paradigm to test whether posterior regions of the brain are of special importance for the processing of spatial prepositions. A cohort of patients with PCA and matched healthy control subjects performed tests of immediate and delayed serial recall to probe working memory for three lexico-semantic word categories. Neuroimaging studies have found that areas close to the site of atrophy and hypometabolism in PCA are engaged during the processing of spatial prepositions (Damasio et al., 2001; Noordzij et al., 2008). Additionally, our previous study using a lexical decision test found a spatial prepositions processing deficit in patients with PCA (Shebani et al., 2017). Based on these previous findings, we hypothesized that the region of primary atrophy and hypometabolism in PCA is important not only for visuospatial and visuo-perceptual processing, but also for the memory processing of words that rely on spatial cognition. We predicted that lesions in posterior parieto-occipital lobe would lead to a memory impairment for spatial prepositions in PCA patients and tested three specific hypotheses related to this prediction.

Hypothesis 1, derived from the semantic topography model, was that PCA patients would show an impairment in memory performance for spatial prepositions compared to healthy controls. As predicted, the PCA patients’ performance relative to controls was most reduced on words from the spatial preposition category in both ISR and DSR (Figure 2). The PCA group’s poorer performance on spatial prepositions, however, was significant only in DSR as revealed by the interaction of the Group and Word Category factors in the between-group analysis and planned comparisons showing a highly significant word category difference in the two group’s performance on spatial prepositions, with PCA patients making 63% more errors than healthy controls. This observation of a deficit in memory performance for spatial prepositions provides strong support for the semantic topography model.

The second hypothesis was that PCA patients would be more impaired on processing spatial prepositions than on processing number words and, indeed, the PCA patients’ memory performance confirms this. As shown in Figure 2, PCA patients made significantly less errors on number words than on prepositions in both serial recall tasks. These results are in line with the semantic topography model’s prediction regarding the greater involvement of posterior-parietal regions in processing spatial prepositions than in processing number words. However, PCA patients also made less errors on number words compared to function words, which goes against the prediction of the semantic topography model. While the word categories in the present study were closely matched for a number of psycholinguistic and semantic variables known to affect word processing, it was not possible to match the word categories for concreteness and imageability, both of which are rated higher for number words. Therefore, the better PCA performance on number words in the present study could also be explained by effects of word concreteness and imageability, especially as the two semantic variables have been found to have an influence on serial recall (e.g., Bourassa and Besner, 1994; Walker and Hulme, 1999; Majerus and Van der Linden, 2003; Tse and Altarriba, 2007; Caplan et al., 2015). Another possibility is that number word lists are simply easier to recall since we often need to remember or recite number strings in our daily life (e.g., phone numbers, addresses), whereas we do not for spatial prepositions and function words.

Hypothesis 3 was that PCA patients would show a stronger deficit for spatial prepositions in DSR than in ISR. This prediction was also supported by the results as revealed by planned comparisons showing significantly more errors on spatial prepositions in DSR compared to ISR for the PCA group (Figure 3). The fact that no other significant word category differences were found between the two memory tasks indicates that the delay in DSR had a damaging effect on the PCA patients’ memory performance for spatial prepositions only. As verbal stimuli are maintained in working memory not only through rehearsal strategies (Baddeley, 1986, 2003) but also through the mechanism of attentional refreshing (Cowan, 2005; Camos et al., 2009; Camos and Barrouillet, 2014), in order for words to be retained in working memory, the semantic representations of the words need to be constantly refreshed. If the cortical areas affected in PCA indeed make a necessary contribution to the semantic processing of words with spatial meaning and since memory traces decay with time in working memory, then it would be expected that the delay in DSR would have a detrimental effect on the retention of spatial prepositions as patients will struggle to keep the semantic representations of the words refreshed during memory maintenance, leading to errors. Our observation of a dramatic increase in number of errors on spatial prepositions in DSR, while performance on number words and function words remained more or less the same, is consistent with this.

PCA patients made a large number of errors on function words in the present study, especially in ISR where the number of errors on function words and spatial prepositions was almost the same. According to the semantic topography model, the representation and processing of function words is restricted to perisylvian regions and reduced PCA memory performance was not expected for this word category. The large number of errors made by PCA patients on function words, however, may be due to effects of word concreteness and imageability. Function words used in the present study were rated very low on both semantic variables. This is not surprising since function words such as *hence* and *shall*, which lack semantic properties, are less conducive to the formation of mental images than, for example, spatial prepositions such as *beneath* and *above*, which more easily evoke a mental image (i.e., of the spatial relation to which they refer). As mentioned above, an influence of concreteness and imageability has been reported in a number of serial recall studies (Walker and Hulme, 1999; Majerus and Van der Linden, 2003; Tse and Altarriba, 2007). Therefore, a limitation of the present study is that it was not possible to match for these semantic variables across the three word categories and some caution is required when interpreting these results. It is noteworthy, however, that despite words from the spatial prepositions category being significantly more concrete and imageable than function words which should lead to enhanced recall of spatial prepositions relative to function words, PCA patients made more errors on spatial prepositions than on function words in DSR.

As significant numbers of errors arising from posterior cortical lesions were made on spatial prepositions, the present study suggests that parieto-occipital cortex makes an important contribution to the processing of spatial prepositions. As aforementioned, an impairment of spatial prepositions relative to number words in PCA patients was demonstrated in a previous study using a lexical decision test (Shebani et al., 2017). The present results, therefore, constitute an important replication of this finding using an entirely different paradigm. That an impairment for spatial prepositions in PCA is present across paradigms and when using very different tasks (lexical decision/serial recall) demonstrates the reliability of this finding and strengthens the argument that parieto-occipital regions are pertinent for processing words with spatial meaning.

The striking impairment for spatial prepositions in the PCA patients’ serial recall performance documented here is consistent with previous reports of processing impairments for words with spatial meaning in patients with PCA (Gonzalez et al., 2019) and in patients with lesioned parietal regions (Tranel and Kemmerer, 2004). Our results also sit well with neuroimaging findings of activated parietal regions during the naming (Damasio et al., 2001) and processing (Noordzij et al., 2008) of spatial prepositions, casting doubt on the possibility that activation in these parietal regions merely reflects imagery processes and is irrelevant for semantic processing (Machery, 2007; Mahon and Caramazza, 2008).

The present results fit well into theoretical frameworks which assume that concepts and word meaning are essentially grounded in sensory and motor systems of the brain (Barsalou, 1999, 2008; Pulvermüller, 2005). Consistent with the semantic topography model, our results demonstrate that sensory regions of the brain that process visuo-spatial information constitute an integral part of the semantic processing of words with spatial meaning. Our findings, therefore, are best explained in terms of distributed semantic circuits in which neurons in modality-specific sensorimotor regions play a functional role in semantic word processing (Pulvermüller, 1999). That is not to say that other brain systems do not have a part to play in conceptual semantic processing. Indeed our results are not incompatible with the role of an amodal semantic ‘hub’ located in anterior temporal lobe for integrating semantic information from modality-specific regions (Patterson et al., 2007; Lambon Ralph et al., 2017). As has been demonstrated in previous studies of neurological patients (Pulvermüller et al., 2010; Shebani et al., 2017), semantic word processing appears to involve cortical circuits distributed over sensorimotor and perisylvian areas as well as amodal semantic convergence zones.

## Conclusion

The degree to which different brain regions contribute to processing word meaning and, in particular, whether sensory and motor regions play a necessary role in processing specific categories of words has generated lively debate recently. The results reported here of a selective and striking memory impairment in patients with PCA point to the functional relevance of parieto-occipital cortex for the processing of spatial prepositions. The present findings, therefore, provide support to grounded theories of semantic processing in general and to the semantic topography model in particular.

## Data Availability Statement

The raw data supporting the conclusions of this article will be made available by the authors, without undue reservation.

## Ethics Statement

The studies involving human participants were reviewed and approved by the Cambridge Local Research Ethics Committee. The patients/participants provided their written informed consent to participate in this study.

## Author Contributions

All authors contributed to the conception and design of the study. ZS collected the data, performed the data analyses and drafted the manuscript. All authors contributed to the article and approved the submitted version.

## Conflict of Interest

The authors declare that the research was conducted in the absence of any commercial or financial relationships that could be construed as a potential conflict of interest.

## Publisher’s Note

All claims expressed in this article are solely those of the authors and do not necessarily represent those of their affiliated organizations, or those of the publisher, the editors and the reviewers. Any product that may be evaluated in this article, or claim that may be made by its manufacturer, is not guaranteed or endorsed by the publisher.
